# Privacy Concerns and Information Sharing: The Perspective of the U-Shaped Curve

**DOI:** 10.3389/fpsyg.2022.771278

**Published:** 2022-05-10

**Authors:** Chien-Lung Hsu, Yi-Chuan Liao, Ching-Wen Lee, Lin Kun Chan

**Affiliations:** ^1^Department of International Business Administration, Chinese Culture University, Taipei, Taiwan; ^2^School of Business, Macau University of Science and Technology, Taipa, Macao SAR, China

**Keywords:** privacy concern, information sharing, social network sites, personal motivation, system evaluation factor

## Abstract

Privacy concerns are a key predictor of information sharing, yet some critical issues remain unclear. Based on social capital theory, this study argues that the relationship between privacy concerns and information sharing is a U-shaped curve. Users with privacy concerns would not share their private information; however, such users would eventually share their information as long as they trust the website and its members. Furthermore, this study provides a contingency perspective, suggesting that the curvilinear relationship between privacy concerns and information sharing varies with the system evaluation perception and personal motivation levels. The results show that at a high level of system evaluation, the relationship between privacy concerns squared and information sharing is non-significant. In contrast, at a low level of system evaluation, there is a U-shaped relationship between privacy concerns and information sharing. Regarding motivation, the results were congruent with our expectations.

## Introduction

Since the advent of the Internet, people have changed the way they interact; the use of social network sites (SNSs), such as Facebook, Twitter, and Instagram, in particular, is growing at a surprising rate. These sites rely on users' participation and contributions to make the service useful and successful. Thus, users' voluntary participation in SNSs is vital. Moreover, SNS users create accounts to discover other people with similar interests or experiences; this requires them to share certain personal information with both friends and strangers or to establish business contacts. While an increasing number of users have joined various SNSs, their privacy concerns persist (Xu et al., 2011; Mahmoodi et al., 2018; Hong and Oh, 2020). Certainly, with the increased use of the Internet, social networking, and other forms of information sharing (IS), privacy concerns remain a topic of much research and discussion (Bergström, 2015; Choi, 2016; Kim, 2016). Thus, information privacy has become an urgent issue for emerging technologies (Aloudat et al., 2014).

Previous research has shown that privacy concerns (PCs) have a negative effect on information system usage (e.g., Osatuyi, 2015; Dhir et al., 2017). However, some scholars argue that users remain willing to disclose information on SNSs that provide interesting and relevant information or in cases that lead to instantaneous problem-solving (Magedanz and Simões, 2009; Dienlin and Trepte, 2015), terming this phenomenon the “privacy paradox.” Thus, in our study, we posit that there is a U-shaped relationship between privacy concerns and information sharing on SNSs.

Furthermore, to understand the phenomenon of privacy concerns regarding information sharing on SNSs, we propose that, due to system evaluation variables and different motivations of SNSs users, the U-shaped relationship between privacy concerns and information sharing on SNSs varies. Based on privacy calculus theory, privacy concerns cannot be viewed as absolute (Princi and Krämer, 2020). The calculus perspective on privacy addresses the joint effects of the perceived benefits and risks associated with privacy perceptions and privacy-protective behaviors (Pentina et al., 2016). As such, in this study, we specify the personal motivations (PMs) and system evaluation factors (SEFs) of SNS users to understand the way users assess value with different motivations and systems (Kang and Shin, 2021). If the perceived benefit outweighs the risk, users with high privacy concerns would share more information. In such a situation, the curvilinear linkage between privacy concerns and information sharing differs; that is, at a high level of a moderator, the U-shaped relationship becomes less pronounced. In contrast, at a low level of a moderator, the U-shaped relationship between privacy concerns and information sharing appears more significant.

This study attempts to make several contributions to the literature. First, this study asserts that privacy concerns are a critical factor worth investigating. We address the fact that individual concerns for information privacy influence users' behaviors on SNSs. It is important to understand privacy concerns beyond binary decisions to withhold or disclose information (Osatuyi, 2015; Lutz and Tamò-Larrieux, 2021). Moreover, the theory argument in the privacy paradox and inconsistent empirical results provide us with a research objective to reconcile the inconclusive arguments. To our knowledge, little research has focused on the non-linear relationship between privacy concerns and information sharing, and no evidence supports the hypothesis that this curvilinear linkage varies with system evaluation perceptions and motivations. As such, this study explores these issues in the personal perception and social behavior literature by verifying the U-shaped curve in the relationship between privacy concerns and information sharing on SNSs.

Second, we provide a contingent view that this curvilinear relationship is affected by system evaluation perceptions and personal motivation factors. This study discusses firms' system factors and individual factors to provide a more holistic view and insights regarding the proposed U-shaped variation in reality.

The remainder of this paper is structured as follows. In the next section, we review some of the literature regarding information sharing, privacy concerns, personal motivation, and system evaluation factors of SNSs. We then present our research model and hypotheses. The following section describes the methodology used to verify the hypotheses. The next section includes the analysis and discussion of the results, followed by the theoretical contributions of this study and a discussion of the managerial implications. After describing the limitations of this study and making suggestions for further research, we present our conclusion.

## Literature Review

### Information Sharing

People build and design social networking sites to provide online services and a platform for social communication and information exchange (Liou et al., 2016). Information sharing, such as exchanging ideas, opinions, feelings, news, and experiences, is one of the necessary functions of SNSs (Ren et al., 2012). Prior research indicates that content gratification was a critical reason for using SNSs, which refers to the extent to which users can access the content of information that was shared in online communities (Syn and Oh, 2015). Park et al. (2014) describe information sharing as a behavior that contributes information to other community members who may need it. This behavior on SNSs is voluntary. Previous research has demonstrated that such voluntary actions rely on pro-social attitudes and rules of organizational ownership (Constant et al., 1994).

### Privacy Concern

Since SNS users are usually required to provide private information on their personal profile, they normally express concerns about their privacy (Lin and Liu, 2012; Lutz and Tamò-Larrieux, 2021). The privacy risk of SNSs has been viewed as a significant factor that affects users' social interactions and usage behaviors (Hajli and Lin, 2016). Smith et al. (1996) have defined information privacy as people's capability to manage when, how, and to what extent their personal information is accessed. Concerns over information privacy may include anxiety about information privacy practices by organizations, such as collecting personal information, unauthorized secondary use of personal information, errors in dealing with personal information, and inappropriate access to personal information (Smith et al., 1996; Hong and Oh, 2020). Privacy concerns increase when users are uninformed about who is collecting their personal information, how SNSs get their information, or for what purposes the information is used (Nowak and Joseph, 1995; Lanier and Saini, 2008). Such negative feelings may make consumers avoid risks regarding information sharing. Therefore, in prior research, scholars generally believe that high privacy concerns are positively correlated with the possibility of taking risk-reducing actions, such as sharing less information. Users' privacy risks are related to their usage behaviors on SNSs. Such privacy risks have been proven to affect people's psychological perceptions and intentions to use information technology (Van Slyke et al., 2006).

### Personal Motivations

Motivation is the intention or driving force by which people reach their desired goals. It has been critical in understanding participation in online social networking. The tripartite perspectives of utilitarian, hedonic, and social dimensions were first used to assess users' motivation (Rintamäki et al., 2006). For instance, utilitarian motivation arises from rational and goal-oriented perspectives of sharing information to accomplish a mission or goal (Mikalef et al., 2013). Hedonic motivation is expressed as delight, enjoyment, and experiences obtained from sharing (Mikalef et al., 2013), whereas social motivation stems from a hunger to earn public recognition. Social motivation aims at seeking the socially recognized or strengthened social self-concept created by SNSs (Yang and Lin, 2016). On SNSs, people may share information to work with others to fulfill common targets, obtain advantages of learning or gaining knowledge, and establish relationships with one another. Without motivation, however, a person easily loses interest and will probably give up the task. Motivation is not static but dynamically changing and evolving, influenced by the conditions or state in which the actions occur (Syn and Oh, 2015).

### System Evaluation Variables

On social networking sites, users are both suppliers and consumers of information and services. Hence, the system qualities of SNSs are critical elements impacting the success of websites. Many studies have investigated the relationship between web quality and user consent in the web context. Most of them indicated that web quality positively influenced user beliefs of perceived helpfulness and perceived ease of use. In this study, we apply two system evaluation variables, namely perceived web information quality and website design appeal. Perceived web information quality is defined as the currency, completeness, reliability, and relevance of the website's content (Bansal and Zahedi, 2015). Unlike regular websites, information on SNSs is undependable as it may be provided by an unknown individual. Hence, the information quality of SNSs is a significant issue for scrutiny. The design appeal of the website may be defined as the visual presentation and the configuration of the website and serves as a source of attractiveness (Bansal and Zahedi, 2015). As the volume of information published daily on SNSs is high, an easy-to-use interface facilitates users in their search process and reduces information processing costs (Zheng et al., 2013).

## Research Method And Hypotheses

### Effect of Privacy Concern on Information Sharing

This study analyzes the U-shaped relationship between privacy concerns and information sharing on SNSs and investigates the moderating effect of system evaluation and users' personal motivations on this curvilinear linkage. First, we assume that there is a U-shaped relationship between privacy concerns and information sharing on SNSs. Former studies argue that if users have more serious privacy concerns, they are unlikely to share their information on SNSs. Wu et al. (2012) stated that online privacy concerns result in unwillingness to provide personal information online, refusal of e-commerce, or even unwillingness to use the Internet. On the other hand, some studies have indicated that the negative relationship between privacy concerns and SNS use or information sharing is weak or lacking. For instance, according to Acquisti and Gross's (2006) survey conducted on student Facebook users, even the privacy-conscious individual revealed a significant amount of private information, which means privacy concerns might have a different impact on the social network. One possible explanation for this discrepancy in the impact of privacy concerns on SNS use or information disclosure is the user's trust in the website owners and other users because SNSs appear to offer a secure platform for users' interaction (Hanifah et al., 2021). Initially, users display concern regarding privacy on the virtual platform as they observe the other members' activities on the website. This study argues that a deeper concern about privacy correlates with a greater tendency to observe SNSs. The privacy-conscious users might gradually interact with other users, such as discussing specific affairs or news. When users increase interaction with other members, they can become more familiar with the website, thereby increasing the possibility of sharing their information (Princi and Krämer, 2020). Krasnova et al. (2010) reiterate this perspective that, although privacy risk is a crucial barrier to self-disclosure, this risk can be mitigated by the user's trust on the website. Once trust is established, users have a higher tendency to share their personal information. Therefore, this study assumes a U-shaped relationship between privacy concerns and information sharing on SNSs.

H1: The relationship between privacy concerns and information sharing is a U-shaped curve.

### Moderating Role of System Evaluation Variables

Bansal and Zahedi (2015) pointed out that users may rely more on peripheral elements to evaluate the site's trustworthiness if they lack the necessary motivation for deeper involvement. People can rely on simple inspections of sketchy features or information quality to evaluate the reliability of a website (Kim and Benbasat, 2003). Nicolaou and McKnight (2006) indicated that users' feelings about features of information such as adequacy, completeness, currency, and timeliness are positively associated with trust. Therefore, if a user perceives a website of poor information quality, he/she will also distrust the reliability of the website regarding other aspects of service, including its capability to secure private information (Jahanshahi and Brem, 2017). Moreover, the website's design appearance related to its visual presentation and structure can attract users. A professionally designed and attractive website reflects the owner's credibility and professionalism (Wells et al., 2011), and it also serves as a representation of the website's quality and influences users' belief and confidence (Wakefield et al., 2004). Hence, when users feel that a certain SNS provides helpful information, such as an informative link or advertisement, it raises their motivation to resume employing that website. In this situation, users may get information from their peers concerning the reliability of the website, which reduces the users' uncertainties. Due to a lack of system evaluation perception, users have fewer reference objects for developing their beliefs. This may cause the U-shape relationship to become less clear between privacy concerns and information sharing. Without useful sources, the user cannot keep using the website, and the uncertainties will remain. In addition, an unappealing design conveys an image of unprofessionalism, implying a lack of credibility and resulting in decreased information sharing. As a result, the relationship between privacy concerns and information sharing has a steeper negative slope. That is, the U-shaped relationship between privacy concern and information sharing looks more significant under the degree of low system evaluation perceptions.

Thus, we propose that the relationship between privacy concerns and information sharing will differ for users with different system evaluation perceptions.

H2: System evaluation variables negatively moderate the U-shaped relationship between privacy concerns and information sharing. That is, a U-shaped relationship between PC^2^ and IS decreases as system evaluation variables increase.

### Moderating Role of Personal Motivations

Our study incorporates Oh's (Oh, 2012) 10 motivation factors, namely enjoyment, efficacy, learning, personal gain, altruism, empathy, community interest, social engagement, reputation, and reciprocity. We use these factors to identify and examine the motivations for information sharing among SNS users. The term “*efficacy*” refers to the user's perceived capability to perform and complete tasks (Bandura, 1997). When users presume that they are proficient in finding information that others might relish or find practical, they are likely motivated to share it. One of the major factors influencing users to continue using SNSs, especially in the adoption stage, is learning. Moreover, Zhao and Rosson (2009) discovered that people use SNSs to seek aid, guidance, and ideas from others. Thus, it would motivate users to search for information and share it with others to gain knowledge about something from others. Personal gain means that the users believe they will benefit from sharing information with others. It can also be viewed as an external reward. Enjoyment means that when users participate in and contribute to SNS communities, they are likely for entertainment. Social media users like to share information for entertainment, amusement, or to kill time (Quan-Haase and Young, 2010). Altruism means that when users become familiar with SNSs, they like to engage and contribute and desire to spend time and effort sharing information with others without any expectation of rewards (Oh, 2012). Empathy refers to the fact that users provide social and emotional support to others (Oh, 2012). Reciprocity refers to the desire of users who have received support or help from others to return the favor to others in the community. Community interest means that by sharing information, users might rally a group of people around a common cause, foster community identification, and stimulate a variety of activities to support the community. Social engagement means that when users desire to connect with others, they might be motivated to communicate with others through sharing information. Reputation means that users gain popularity by sharing information on SNSs. Pai and Arnott (2013) indicated that the users' desire for popularity is inextricably linked to gaining respect and the value of self-esteem from others in a community. Because of the different motivations, users would have a social or non-social effect on their involvement on the SNSs. Thus, we demonstrate that the relationship between privacy concerns and information sharing will differ for users with different motivations.

Zhao and Lu (2012) used network externalities and the motivation theory to analyze what factors affect SNS users, such as perceived extrinsic and intrinsic benefits and network externalities, including direct (number of members), peer (number of peers), and indirect (perceived complementarity) network externalities. Furthermore, according to social cognitive theory, people implement certain behaviors, partially influenced by dynamic and mutual interactions between their personal and cognitive factors (e.g., self-efficacy and outcome expectations) and social environments (e.g., social systems and social networks) (Bandura, 1997; Kang and Shin, 2021). During the process, users driven by a goal-directed perspective choose and apply media that provide them with a way to satisfy a large variety of needs (Katz et al., 1974). Thus, achieving extrinsic motivations such as efficacy, learning, and personal gain can optimize users' benefits during the information sharing process with others (Bock et al., 2005). After evaluating the advantage and privacy cost, users might exhibit increased behavior on SNS information sharing in which people are more likely to undertake that system when they are extrinsically motivated to do so (Park et al., 2014). However, users evaluate their interactions with SNSs based on intrinsic motivation such as pleasure, altruism, empathy, and reciprocity, determining their degree of “feeling good.” When people are in a state of flow, they will concentrate on the current activity and filter out irrelevant perceptions. This causes them to fully focus on the activities (Hung et al., 2016). Because of the hedonic feeling, the U-shaped relationship between privacy concerns and information sharing is less significant. Choi (2016) stated that people prefer closed-type SNSs because they allow them to share information only with similar groups to which they have a sense of belonging. Thus, even the most privacy-conscious people share information if they believe the SNS is reliable and safe. In addition, Wu et al. (2012) indicated that virtual community members with shared common values tend to sustain relationships in the community, and these shared values heighten trust and positive expectations in the virtual community. Users have trust expectations when community members share the same language and thoughts with similar interests and values to communicate with each other on the SNSs while interacting. This means that social motivations such as community interest, social engagement, and reputation encouraged trust among users regarding how they use personal information. Thus, the negative slope of the relationship between privacy concerns and information sharing is less significant.

H3: Personal motivation variables negatively moderate the U-shaped relationship between privacy concerns and information sharing. That is, a U-shaped relationship between PC^2^ and IS decreases as personal motivations increase.

**Figure 1:**
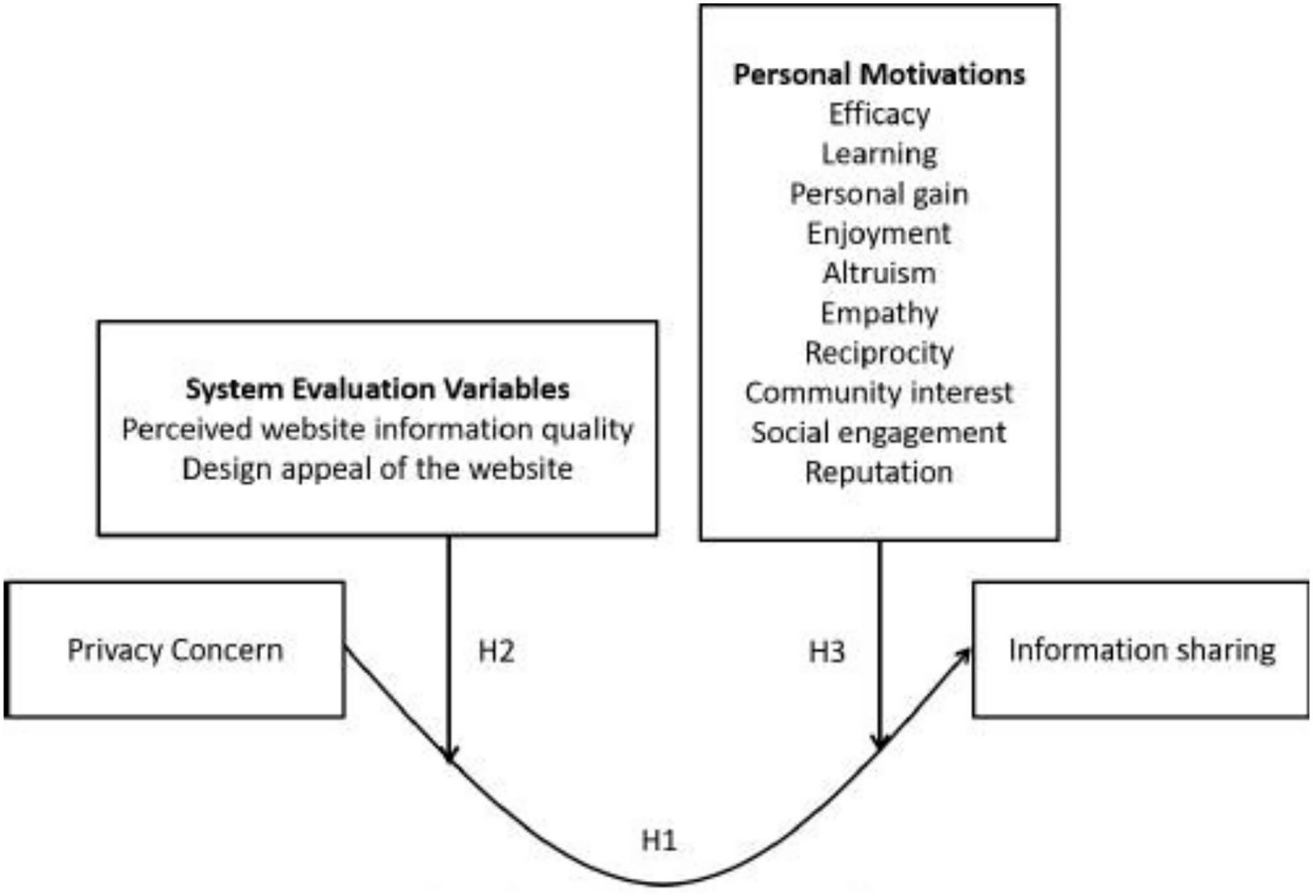
Research framework.

Figure 1 presents the theoretical framework of this study.

## Research Methods

### Model


(1) $$\begin{array}{l} IS=B_{0}+B_{1}PC+B_{2}PM+B_{3}SEV+B_{4}PC^{*}PM\\ \;+B_{5}PC^{*}SEV+B_{6}PC^{2}+B_{7}PC^{2*}PM\\ \;+B_{8}PC^{2*}SEV. \end{array}$$

Note: PC, privacy concern; PM, personal motivations; SEV, system evaluation variables; IS, information sharing; X'B, a linear combination of the control variables.

### Data Analysis and Samples

The measure was originally written in English, and we translated it into Chinese using the following procedures. We hired two graduate students from a business school: One is a PhD student, and the other is pursuing a master's degree. They translated our measure into Chinese. Two assistant professors with at least 3 years of experience in research and publications in high-quality journals were involved in the process to ensure the content validity and appropriateness of the measures in our research context. The data used in this research were collected via an online sampling survey hosted at my3q.com (http://www.my3q.com). The respondents were Taiwanese SNS users of websites such as Facebook (*n* = 193), Instagram (*n* = 59), Twitter (*n* = 3), and PTT^1^ (*n* = 24). To encourage participation, the respondents were offered the opportunity to win a lottery-based prize (7-Eleven i-cash or Family-Mart gift card). The respondents were asked to evaluate the items in the questionnaire based on their behaviors. On the whole, 279 individuals participated in this study (47.7% men and 52.3% women; median age = 21–30 [48.8%]). In terms of education, most of the participants were undergraduates (60.9%), followed by postgraduates (31.5%).

### Measure

We applied previously published measurement items and modified them to fit our study. Furthermore, we drew items to measure the constructs of information sharing (Oh, 2012), and adapted that for perceived information quality (McKinney et al., 2002). Moreover, we applied measurement items from Bock et al. (2005) to construct the design appeal of the website and privacy concerns. In our study, the system evaluation factor includes the perceived information quality and design appeal of the website. The motivations were adapted from Oh (2012). We used a five-point Likert scale (ranging from 1 “strongly disagree” to 5 “strongly agree”) in our questionnaire. The mean and standard deviation are demonstrated in Table 1.

**Table 1:** Means, standard deviations, and correlations (*N* = 279).

**Variable**	**PC**	**SEV**	**PM**	**IS**	**Gender**	**Age**	**Edu**	**Length**
1	1							
2	0.16	1						
3	0.27	0.65	1					
4	0.08	0.51	0.73	1				
5	0.05	0.14	0.20	0.14	1			
6	0.35	0.31	0.38	0.27	0.09	1		
7	0.06	0.10	0.07	0.07	0.26	0.03	1	
8	0.02	0.21	0.28	0.23	0.15	0.11	0.01	1
Mean	3.64	3.79	3.64	3.77	0.52	3.43	3.25	5.00
SD	0.77	0.57	0.66	0.67	0.50	1.66	0.81	1.88

*PC, privacy concern; SEV, system evaluation variables; PM, privacy motivations; IS, information sharing; Length, spend time on internet in a day*.

### Reliability and Validity

Before examining our hypotheses, our study utilized several steps to ensure the reliability and validity of our model. For reliability, the constructs were evaluated with Cronbach's α and composite reliabilities (CRs). The statistical results showed that all Cronbach's α and CRs exceeded the threshold of 0.7, in accordance with Fornell and Larcker's recommendations (Fornell and Larcker, 1981). According to Nunnally (1978), our measures have adequate internal consistency and reliability.

Furthermore, we conducted the confirmatory analysis (CFA) to evaluate validity. The CFA of a four-construct model showed that the whole model conforms well to the data (χ^2^/d.f = 4023.01/1,024 = 3.93, CFI = 0.94, IFI = 0.94, RFI = 0.92). All items loaded significantly on the corresponding latent construct, with fair lambda values ranging from 0.50 to 0.92 (*p* < 0.01). All average variances extracted (AVE) were higher than 0.5. These results indicate the appropriate convergent validity (Fornell and Larcker, 1981; Bagozzi and Yi, 1988). Discriminant validity was adopted via two approaches. First, we used a series of chi-square tests for all constructs in pairs to determine whether the unrestricted model is significantly higher than the restricted model: in other words, once to constrain the correlation between the constructs to 1 and the other to free the parameter (Podsakoff and Organ, 1986). All combinations yielded a greater significant value (Δχ^2^_(1)_ = 3.84 at the 5% significance level). Second, we assessed whether the confidence interval (±2 standard errors) around the correlation evaluation between two constructs includes 1. All the confidence intervals of pairs do not include 1. Third, this study followed the heterotrait–monotrait (HTMT) ratio of the correlations proposed by Henseler et al. (2015) and Voorhees et al., 2016. The threshold value for the ratio was 0.90 for correlations between the constructs (Henseler et al., 2015). In the present study, our values range from 0.08 to 0.81, which falls below the 0.9 benchmark, as shown in Table 2. In summary, several tests support the discriminant validity of our study. This study further used statistical methods to assess the common method variance (Podsakoff et al., 2003). We performed Harman's single-factor test. Our results indicated that a prominently common method did not exist in the data (Podsakoff and Organ, 1986). To sum up, the results indicate there is no common method variance in this study.

**Table 2:** Discriminant validity: heterotrait–monotrait (HTMT).

	**PC**	**SEV**	**PM**	**IS**
1				
2	0.18			
3	0.29	0.72		
4	0.08	0.62	0.81	

*PC, privacy concern; SEV, system evaluation variables; PM, privacy motivations; IS, information sharing*.

## Results

In our study, we used the hierarchical regression model, which is widely adopted to test the examination of association for evidence of a non-linear relationship to verify our hypotheses. Before estimating our hypotheses, we first standardized the measures of variables, including control, explanatory, and criterion variables, and then formed the cross-product terms and quadratic terms (Friedrich, 1982; Aiken and West, 1991). The variance inflation factor (VIF) for all the regression models was under 10, indicating unproblematic multicollinearity. According to our hypotheses, Model 1 entered the control variables that included SNS category, gender, age, education, and website use (length). Model 2 added the variables that included privacy concerns and moderators. Model 3 added the quadratic term of customer engagement to the regression equation to test the curvilinear relationship. Models 4 and 5 added the interaction terms between the squared privacy concern and moderator variables (SEV and PM).

In Table 3, Model 1 indicates that information sharing is not affected by SNS category, gender, or education level. Nonetheless, with an increase in age and duration, users show greater tendencies to share information. With an increase in the user's age, the number of friends increases as well, resulting in increased interaction with those friends and information sharing. Regarding the length of usage, it is evident that users are more likely to share their information once they continue using the website. In Model 2, our results indicate that people concerned about their privacy on the Internet are less willing to share information (ß = −0.14, *p* < 0.01). Moreover, the system evaluation factor has no impact on information sharing directly (ß = 0.06, *p* > 0.05), but motivation has a significant effect (ß = 0.742, *p* < 0.01). This is consistent with prior viewpoints that stress users' motivation to be involved in their community and share their thoughts or ideas. In Model 3, our results indicated that our square term was positive, but non-significant (ß = 0.02, *p* > 0.05). Hence, the results do not verify our hypothesis 1 that the relationship between privacy concerns and information sharing is U-shaped. To test our moderating arguments, Model 4 demonstrates that the regression coefficient of the interaction term is positive, but non-significant between PC and system and motivation (ß = 0.03, *p* > 0.05 and ß = 0.02, *p* > 0.05, respectively). Model 5 demonstrates that the estimated coefficient of the interaction term is negatively significant between PC^2^ and information sharing (ß = −0.11, *p* < 0.01). The results do not sustain our hypothesis 2, which indicates system evaluation factors positively moderate the U-shaped relationship between PC and information sharing. Instead, the results reveal that system evaluation factors negatively impact the curvilinear relationship between PC and information sharing. In addition, Model 5 also demonstrates that the regression coefficient of the interaction term is significantly positive between PC^2^ and motivation (ß = 0.14, *p* < 0.01), supporting our hypothesis 3 that motivation positively moderates the U-shaped relationship between PC and information sharing.

**Table 3:** Results of hierarchical moderated regression analyses.

**Variables**	**Model 1**	**Model 2**	**Model 3**	**Model 4**	**Model 5**	**VIF**
Categroy	Controlled	Controlled	Controlled	Controlled	Controlled	
Gender	0.15 (1.30)	−0.04 (−0.50)	−0.05 (−0.55)	−0.06 (−0.66)	−0.06 (−0.63)	1.18
Age	0.15^**^ (4.23)	0.02 (0.72)	0.02 (0.69)	0.01 (0.44)	0.02 (0.53)	1.39
Edu	0.06 (0.81)	0.03 (0.59)	0.03 (0.61)	0.04 (0.70)	0.04 (0.75)	1.10
Length	0.10^**^ (3.10)	0.02 (0.73)	0.02 (0.65)	0.01 (0.55)	0.01 (0.53)	1.14
PC		−0.14^**^ (−3.20)	-0.13^**^ (−2.54)	−0.13 (−2.45)	−0.13^**^ (−2.53)	1.62
SEV		0.06 (1.02)	0.06 (0.99)	0.06 (1.13)	0.16^**^ (2.34)	2.96
PM		0.74^**^ (12.08)	0.74^**^ (12.80)	0.74^**^ (12.50)	0.60^**^ (7.74)	3.74
PC^2^			0.02 (0.56)	0.01 (0.44)	0.05 (1.43)	1.77
PC^*^SEV				0.03 (0.61)	−0.02 (−0.04)	2.37
PC^*^PM				0.02 (0.32)	0.10 (1.52)	2.66
PC^2^^*^SEV					−0.11^**^ (−2.53)	4.58
PC^2^^*^PM					0.14^**^ (2.82)	5.77
*R* ^2^	0.15	0.56	0.56	0.56	0.58	
F-value	7.92^**^	38.27^**^	34.35^**^	28.64^**^	25.74^**^
F-value for Δ*R*^2^	7.92^**^	84.29	^**^0.31	0.62	4.22^*^

*N = 279*.

* *p < 0.05*,

** *p < 0.01*,

*t-values are in parentheses*.

*Category, different SNS (such as Facebook, Instagram); Length, spend time on internet in a day; PC, privacy concern; SEV, system evaluation variables; PM, privacy motivations; IS, information sharing*.

**Figure 2:**
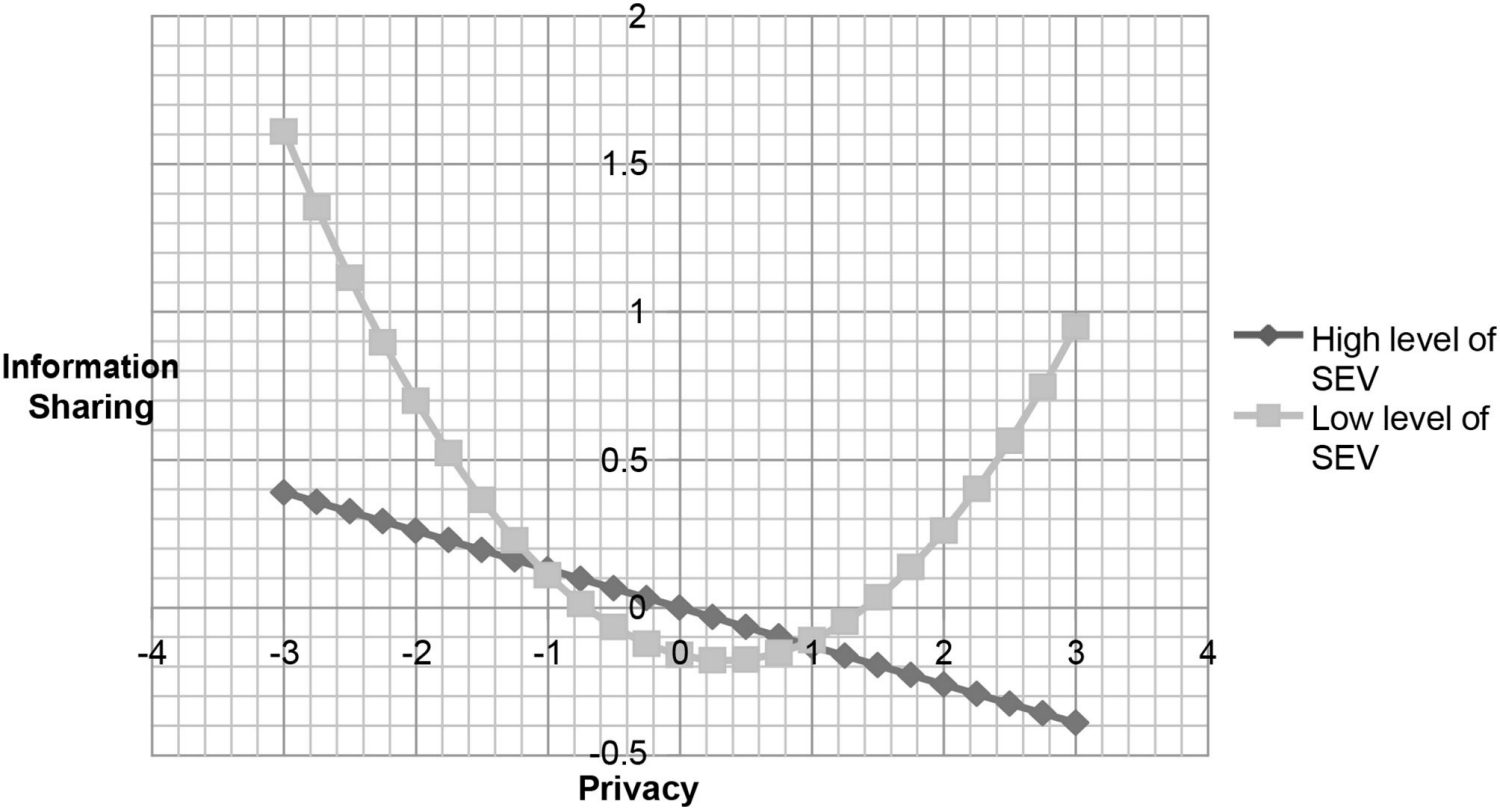
The relationship between privacy concern and information sharing under different level of system evaluation variables.

**Figure 3:**
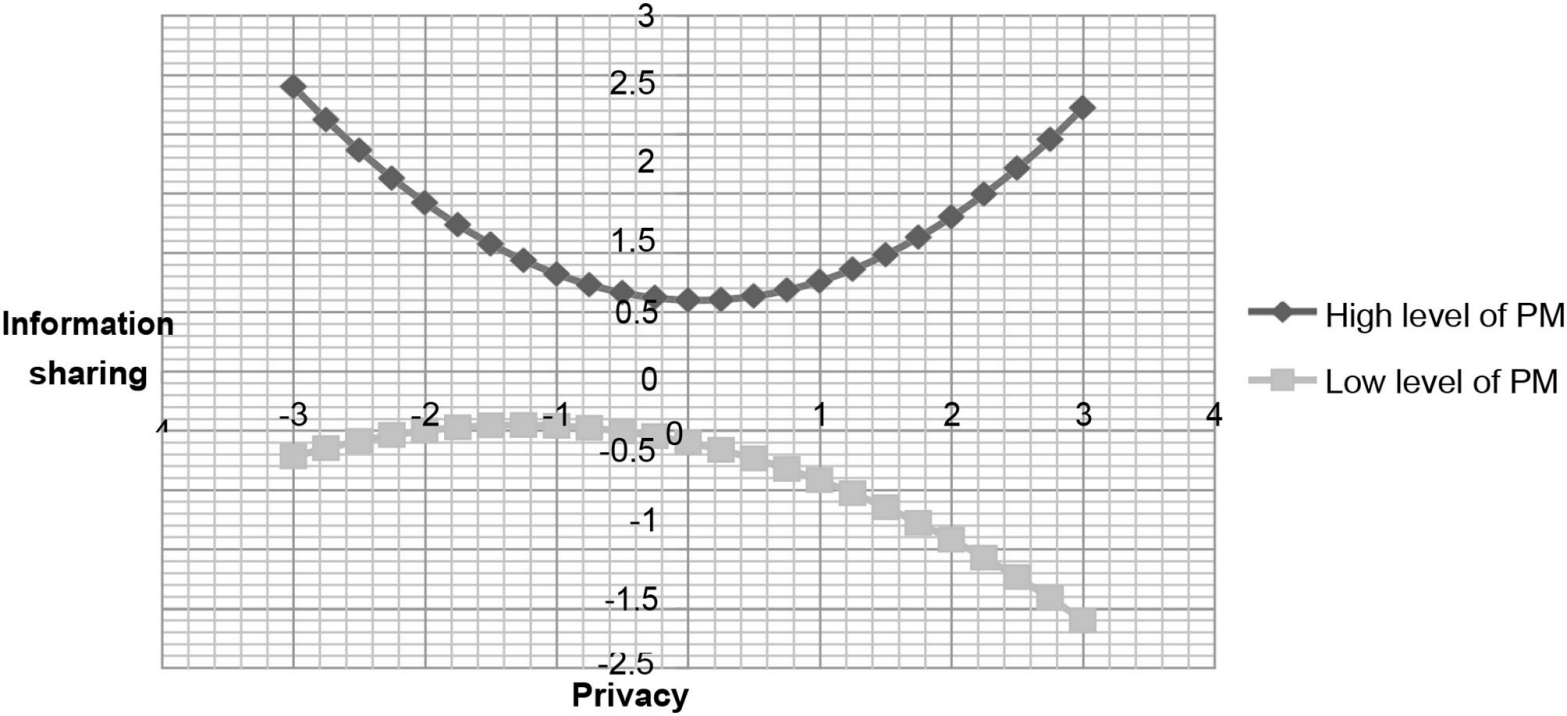
The relationship between privacy concern and information sharing under different level of personal motivation.

To acquire more insight into these effects, we used the procedure previous studies [43] suggested to investigate the essence of the interactions. This *post-hoc* analysis is adopted to assess the significance of regression coefficient estimates for the PC^2^ variable at one deviation above and below the mean of the moderators (i.e., system and motivation). Although hypothesis 2 does not support our assumption, we continue to inspect the negative moderating effect of developing a perception about system evaluation in developing privacy concerns on information sharing. A negative but non-significant relationship was found between privacy concerns squared and information sharing (ß = −0.06, *t* = 1.13, *p* > 0.05) at a high level of system evaluation factor. The results also revealed a positively significant relationship between privacy concerns squared and information sharing (ß = 0.16, *t* = 3.02 *p* < 0.01) at a low level of system evaluation. In addition, a significantly positive relationship was found between privacy concerns squared and information sharing in terms of user motivation (ß = 0.19, *t* = 3.17, *p* < 0.01) at a high level of motivation. At a low level of motivation, the relationship was negative, but non-significant, between PC^2^ and information sharing (ß = −0.09, *t* = 1.5, *p* > 0.05). We plotted the relationship between privacy concerns and information sharing as shown in Figures 2, 3 to examine the slopes for future clarity. These figures indicate that the curvilinear relationship varies with the different system levels and motivation between PC and information sharing.

## Discussion And Implications

Today, SNSs are rapidly replacing traditional modes of communication (face to face), and individuals are increasingly concerned about their privacy, exerting influence over the information they share. Prior studies' arguments and empirical mixed results confuse academic research and practical implications. Thus, this study emphasized that there is a U-shaped relationship between privacy concerns and information sharing on SNSs. This study considered two critical factors: corporation perspective (system evaluation factor) and personal perspective (individual motivation), to influence the U-shaped that we argue. Our study systematically expounded on the users' online information sharing behaviors.

### Theoretical Implications

Echoing prior research, research on information privacy areas should further explore the complex relationship between individuals' concerns for information privacy and their behaviors (e.g., Osatuyi, 2015). This study proposes a curvilinear relationship between privacy concerns and information sharing and includes the moderating effect of different factors on this relationship. This is in contrast to previous studies that argue that people with privacy concerns would not share private information. In addition, some scholars have proposed a positive linear relationship between privacy concerns and information sharing. This study introduces a broader approach to examine the U-shaped relationship between privacy concerns and information sharing. The results of this study are the first to demonstrate that there is no curvilinear relationship between privacy concerns and information sharing. Nevertheless, when system evaluation constructs and motivation are included in this discussion, the relationship between privacy concerns and information sharing does show a curvilinear relationship. This result is consistent with the previous studies, suggesting that involvement in an online community is contingent on different contexts (e.g., Pentina et al., 2016). Interestingly, we observed a non-significant relationship between the square of privacy concerns and information sharing at a high level of system evaluation. Additionally, the results of this study show that there is a U-shaped relationship between privacy concerns and information sharing at a low level of system evaluation.

As far as motivation is concerned, the results confirmed our expectations. At a high level of motivation, initially, people are concerned that their information may be abused; however, after a strong incentive and willingness to interact with the community members or observe the other members' activities, they may develop confidence and trust in the community (Ren et al., 2012). Therefore, once people with strong motivation join the community, their concerns regarding information abuse will decrease, with an increase in information sharing with their community members (Mikalef et al., 2013). On the other hand, at a low level of motivation, initially, people with less incentive may still interact with other members or users and share little information or ideas. However, as their privacy concern increases, they become more sensitive and feel anxious that their information may be abused or leaked by the SNSs. Thus, users become unwilling to share information on SNSs.

### Managerial Implications

We found that under a high level of system evaluation the linear relationship between privacy concerns and information sharing is negatively significant. This may reflect how people view SNS platforms in reality. Although we argue that the design appeal of the websites is correlated with visual presentation and configuration, it is the source of attractiveness (Wakefield et al., 2004). However, as users' privacy concerns increase, they may become aware that their private information may be stored or abused by the SNSs. For example, Facebook admitted that its users' data were leaked to Cambridge Analytica, a consulting firm. Therefore, nowadays, as people evaluate the system with superior functions, such as attractive website designing or offering precise information, they may be concerned about their private information being abused by the SNSs. On the other hand, at a low degree of system evaluation factors, this relationship demonstrates that an increase in information sharing first declines and then increases. This means that, at a low level of system functions, initially, people may worry about their information being leaked, but as they interact with their community members, they may develop a sense of belonging with the community, even if they perceive that a given SNS's function may not be superior to those of other websites. As such, those people may share more information on the given sites. At a high level of motivation, individuals will mitigate the effects of their privacy concerns on information sharing. Business managers may provide users with entertainment or interesting topics to attract participants' involvement and then increase their motivation to share their information.

### Limitations and Future Research

This study has several limitations. First, our study focuses on individuals who are concerned with their privacy and their information sharing behavior; however, different generations may have different privacy concerns and information behaviors (Dhir et al., 2017). Future research can introduce different generations into our framework to explain the interplay among such participants and associated influences on the relationship between privacy concerns and information sharing. Second, we aimed to explain the situation we face in reality; however, conducting a survey may have some limitations. Future research can utilize data from business corporations to ensure that our framework is appropriate.

## Conclusion

In summary, this study contributes to Internet literature in a number of ways. First, this study employs the privacy concern that people have regarding the possibility that their information may be leaked or abused. Although prior studies state that privacy concerns may have a negative impact on sharing information, this study argues that privacy concerns and information sharing may have a curvilinear relationship. We found that the curvilinear relationship is contentment in different contexts. The results indicate that the system evaluation factor may negatively affect the relationship. At a high level of system superiority, users present intense concerns about their privacy, reducing their willingness to share. By contrast, they may believe their information is not abused when the system is at a low level of superiority, and after a certain point, they build the membership and are willing to share information. Second, we propose that motivation is a critical factor in mitigating the negative effect of privacy concerns. Our results verify our argument and may help explain why, even after Facebook admitted its mistake, people continue to share information on SNSs. As such, managers should consider how to prompt users' privacy concerns on sharing.

## Data Availability Statement

The raw data supporting the conclusions of this article will be made available by the authors, without undue reservation.

## Author Contributions

C-LH was responsible for generating ideas, developing the main framework and hypotheses, and in charge of the structure of manuscript. Y-CL was responsible for theorizing the hypotheses, data analysis, and manuscript revisions. C-WL was responsible for organizing, writing, and editing the literature. LC was responsible for data collection and literature review. All authors contributed to the article and approved the submitted version.

## Funding

We would like to thank the Macau University of Science and Technology for Faculty Research Grant awarded to Y-CL (Grant No. FRG-21-029-MSB), and to LC (Grant No. FRG-21-011-MSB) for supporting this research.

## Conflict of Interest

The authors declare that the research was conducted in the absence of any commercial or financial relationships that could be construed as a potential conflict of interest.

## Publisher's Note

All claims expressed in this article are solely those of the authors and do not necessarily represent those of their affiliated organizations, or those of the publisher, the editors and the reviewers. Any product that may be evaluated in this article, or claim that may be made by its manufacturer, is not guaranteed or endorsed by the publisher.

## Footnotes

^1^PTT is a bulletin board system based in Taiwan that was founded on the basis of Taiwan's online academic resources and originated for academic purposes; it remains one of the mainstream online forums in Taiwan.
