# Supplementary material for: Privacy Concerns and Information Sharing: The Perspective of the U-Shaped Curve
Source: Front Psychol. 2022 May 10;13:771278. doi: 10.3389/fpsyg.2022.771278 (PMC9128837; doi:10.3389/fpsyg.2022.771278)
Supplement: Supplementary file 1 [file Data_Sheet_1.docx]

**Appendix**

| Constructs | Measures |
| --- | --- |
| Perceived information quality | - Concerned with issues such as quality, clarity, and relevancy of the information |
|  | - Concerned with the immediacy of the information. |
|  | - Concerned with the degree of consistency, reliability, and accuracy of the information. |
|  | - Evaluates the extent of the information, range of the information, and level of details provided by the website. |
|  | - Users’ assessment of the likelihood that the information will enhance their purchasing decision. |
|  |  |
| Design appeal of the website | - The degree of professionality of the website design. |
|  | - The website’s design quality is high. |
|  | - The website’s layout looks organized. |
|  |  |
| Privacy concern | - Submitting personal information on the Internet is (highly advisable/not advisable at all). |
|  | - Personal information on the Internet, once submitted, (will not be abused at all/will be abused for sure). |
|  | - Personal information on the Internet, once submitted, (will not be stolen at all/could be shared or sold to others). |
|  | - The extent of my concern regarding the misuse of my personal information submitted on the Internet is (very low/very high). |
|  |  |
| Motivation | I share my posts on Facebook/Twitter because… |
|  | - It is fun. |
|  | - It makes me happy. |
|  | - It is my hobby. |
|  | - I feel productive in creating or finding interesting information for others. |
|  | - I feel competent distributing interesting information to others |
|  | - I want to return the favor because I find interesting information from others' wall posts. |
|  | - I believe I can also find information I need from others' wall posts. |
|  | - It may encourage people to "pay it forward" by sharing their information with others. |
|  | - It gives me a feeling of accomplishment. |
|  | - It helps me learn about topics in which I am interested. |
|  | - It helps me learn about information people are interested in. |
|  | - It helps me know about updated information. |
|  | - I want to help others find information that they look for. |
|  | - People should help each other look for information. |
|  | - I enjoy helping others. |
|  | - I empathize with those who have trouble finding the information they need. |
|  | - I empathize with those who have similar information needs as I do. |
|  | - People may not find good information if I do not share wall posts. |
|  | - It helps to promote the topic in which I’m interested. |
|  | - It helps to promote the area in which I have expertise. |
|  | - It helps to promote Facebook/Twitter. |
|  | - It helps me communicate with others. |
|  | - It helps me interact with others better in a community. |
|  | - I feel intergated socially. |
|  | - I’m happy to see the number of my friends increase. |
|  | - I'd like to be recognized as a contributor. |
|  | - My reputation can be built in a community. |
|  |  |
| Information sharing | - I will provide important, valuable, or interesting information to others. |
|  | - I will share my experience or knowledge with others. |
|  | - I will provide information I know when others need help. |
|  | - I will try to offer information obtained from all kinds of channels to others. |
